# 

**Published:** 1855-03

**Authors:** 


### CONTENTS OF NO. III., VOL. II.

ORIGINAL COMMUNICATIONS.

The Seventh Census, &c.—By Prof J. E. Sanborn. . 161
Epidemic Erysipelas—By W. S. Marsh, M. D. . . 171
Lithotomy—Bilaterial Operation—By Prof. Hughes. . 178

DEPARTMENT OF SELECTIONS.

Beverages in Sickness—By L. A. Dugas, M. D. . . 182
Boston Soc. Med. Improvement—By Dr. Morland. . 188
Water Dressing—By Geo. H. Hubbard, M. D. . . 195
Water, in Disease, &c.—By T. C. Rutherford, M. D. . 198
Anæmia of Infancy—By Prof. Mauthner. . . . 200
Effects of Shaving—By E. Sanborn, M. D. . . . 202
Alkalis in Rheumatism—By Geo. Budd, M. D. . . 205
Death from Old Age—By Dr. Green. . . . 208
Gun-Shot Wound of the Brain—By Dr. Smith, . . 209
Denarcotized Opium in Fever—By Dr. Sinn, . . 211
Acids in Disorders of the Bowels, . . . . . 212
Radical Cure of Hydrocele, Without Injections, . . 213
A Substitute for Gum Arabic, . . . . . 214
Decoction of Oats as a Diuretic, . . . . . 215
Ethereal Solution of Nitrate of Silver, . . . . 215
Improved Stethescope, Sore Nipples, &c., . . . 216

EDITORIAL DEPARTMENT.

Sanitary Condition of Western Steamers, . . . 217
Homœopathy Legitimately Developed, . . . . 223
Close of Session and Graduating Exercises, . . . 225
American Med. Association—Next Meeting, . . . 229
Medical Association—Prize Essays, . . . . . 230
Professional Quackery, . . . . . . . 230

BIBLIOGRAPHICAL NOTICES.

Transactions of American Medical Association, . . 233
Positive Medical Agents, . . . . . . . 237
What to Observe at the Bed-side and After Death, . 238
Medical Obituaries, . . . . . . . . 239

